# Using qualitative and community-based engagement approaches to gain access and to develop a culturally appropriate STI prevention intervention for foreign female entertainment workers in Singapore

**DOI:** 10.1186/s12992-018-0358-5

**Published:** 2018-04-16

**Authors:** Raymond Boon Tar Lim, Olive N. Y. Cheung, Dede Kam Tyng Tham, Hanh Hao La, Thein Than Win, Roy Chan, Mee Lian Wong

**Affiliations:** 1 Saw Swee Hock School of Public Health, National University of Singapore, Tahir Foundation Building, 12 Science Drive 2, #10-01, Singapore, Singapore city, 117549 Singapore; 2 Humanitarian Organisation for Migration Economics (HOME), 304 Orchard Road, #06-22, Lucky Plaza, Singapore, Singapore city, 238863 Singapore; 3 Department of Sexually Transmitted Infections Control, National Skin Centre, 31 Kelantan Lane, #01-16, Singapore, Singapore city, 200031 Singapore

**Keywords:** Female entertainment worker, Indirect sex worker, Entertainment establishment, Hard-to-reach, Population size estimation, Census enumeration, Singapore, Human immunodeficiency virus, Sexually transmitted infections, Qualitative inquiry

## Abstract

**Background:**

There is an increasing global movement of foreign female entertainment workers (FEWs), a hard-to-reach population vulnerable to HIV/STIs. This paper described the needs assessment phase before intervention implementation where the socio-organisation, sexual risk behaviours and access to health services of foreign FEWs in Singapore were explored. We also highlighted how qualitative inquiry, census enumeration technique and community-based engagement approaches were used to gain access and to develop a culturally appropriate STI prevention intervention.

**Methods:**

In-depth interviews, observations, informal conversational interviews, mystery client and critical incident technique were used. We estimated the size of FEW population using the census enumeration technique. The findings were used to inform intervention development and implementation.

**Results:**

We estimated 376 Vietnamese and 330 Thai FEWs in 2 geographical sites where they operated in Singapore. Their reasons for non-condom use included misconceptions on the transmission and consequences of STI/HIV, low risk perception of contracting HIV/STI from paid/casual partner, lack of skills to negotiate or to persuade partner to use condom, unavailability of condoms in entertainment establishments and fear of the police using condom as circumstantial evidence. They faced difficulties in accessing health services due to fear of identity exposure, stigmatisation, cost and language differences. To develop the intervention, we involved FEWs and peer educators, and ensured that the intervention was non-stigmatising and met their needs. To foster their participation, we used culturally-responsive recruitment strategies, and ensured that the trial was anonymous and acceptable to the FEWs. These strategies were effective as we achieved a participation rate of 90.3%, a follow-up rate of 70.5% for the comparison and 66.8% for the intervention group. The interventions group reported a significant increase in consistent condom use with a reduction in STI incidence compared to no significant change in the comparison group.

**Conclusions:**

The qualitative inquiry approaches to gain access, to foster participation and to develop a culturally appropriate intervention, along with the census enumeration technique application to estimate the FEW population sizes has led to successful intervention implementation as well as safer sexual behaviour and STI incidence reduction.

**Trial registration:**

ClinicalTrials.gov, NCT02780986. Registered 23 May 2016 (retrospectively registered).

**Electronic supplementary material:**

The online version of this article (10.1186/s12992-018-0358-5) contains supplementary material, which is available to authorized users.

## Background

Female sex workers (FSWs) are a diverse population operating in different settings such as brothels, streets, online sex forums and entertainment establishments (EEs) [1]. EEs are karaoke lounges, bars, pubs, nightclubs and discotheques providing entertainment activities such as singing, dancing and social drinking [2]. Female entertainment workers (FEWs) working in EEs are often indirect sex workers who sell sex in the EEs to supplement their income [3, 4]. Indirect sex work is illegal in many countries [5]. Due to stigmatisation, discrimination and fear of prosecution, FEWs are a hard-to-reach, ill-defined and mobile population. They are distrustful of outsiders and will engage in all means to avoid the revelation of their identities [6]. Compared to brothels with 100% condom use programme, there is a lack of access to human immunodeficiency virus (HIV)/ sexually transmitted infection (STI) prevention programmes in the EE setting [7, 8]. The prevalence of HIV/STI was higher among FEWs than brothel-based FSWs in a systematic review [9]. As FEWs often face difficulties in accessing preventive or healthcare services, they are vulnerable to HIV/STIs [2, 8, 9].

In recent years, sex work has increasingly shifted from brothels to EEs in Asia [10]. Globalisation, wide income disparities, popularity of adult entertainment and ease of travel have led to an influx of women from countries in Asia such as Thailand, Vietnam, China, Philippines and Indonesia to work in EEs worldwide [10–12]. For example, female Chinese nationals have worked as foreign FEWs in Los Angeles, United States of America (USA) [13]; foreign Chinese and Thai women in Sydney, Australia [14]; foreign Vietnamese and Thai women in San Francisco, USA [15] and in Singapore [8]. Foreign FEWs are more vulnerable to HIV/STIs than their native counterparts as they experience additional barriers that diminished their ability to seek preventive care services. These included their foreign status in the country of work and having to operate in a foreign working environment which is culturally and linguistically different from their native land [2].

This global change in the sex work industry has brought attention to the health needs of these indirect sex workers, particularly foreign FEWs [11]. Preventive interventions need to reach the most active sex workers to reduce their burden of STI/HIV [16]. Only one study pertaining to native sex workers, Benoit et al. [17] was included in a recent systematic review of strategies to reach hard-to-reach population [18]. Benoit and colleagues discussed the usage of qualitative inquiry approaches such as informal conversational interviews and documentary reviews in understanding how a diverse population of sex workers operated in different sectors of the sex industry in a Canadian metropolitan area [17]. The information collected was then used by the researchers to gain access to them for health research.

Qualitative approaches are commonly used in studying social phenomena in hard-to-reach populations, particularly for topics which are intensely private, and sometimes illicit [19]. Examples of approaches used included in-depth interviews (IDIs), observations, ethnographies, focus group discussions (FGDs), along with emerging ones such as mystery client and critical incident techniques [20]. They have contributed to the development, evaluation and refinement of interventions for hard-to-reach populations, particularly for FSWs [19, 21]. For example, IDIs, observations and ethnographies have been frequently used to explore the behavioural, educational and health service needs of FSWs [22] such as their willingness to use STI/HIV preventive services [23], as well as in process evaluation to understand their barriers and facilitators for participating in STI/HIV prevention programmes [24]. However, few studies have described the use of these approaches to gain access and to develop a culturally appropriate intervention for foreign FEWs [25, 26].

The size of these mobile and ill-defined FEWs is often unknown, making them more difficult to access [6]. Various techniques such as census enumeration, capture-recapture, multiplier and respondent-driven sampling have been developed to estimate the size of hard-to-reach populations [27, 28]. They have been used on native FSWs but not on foreign FEWs [29, 30]. Each of these techniques has their own strengths and weaknesses, with no current gold standard [27, 28]. Among these techniques, respondent-driven sampling has been widely used among FSWs, intravenous drug users, men who have sex with men (MSM) and migrants [31]. We used the census enumeration technique instead of respondent-driven sampling for our study because it is more appropriate for populations in which a sampling frame exists [27, 28]. We have conducted observations and found that the foreign FEWs, whom we wanted to gain access to, operated in well-defined EEs in geographically distinct areas. A listing of the EEs in these areas is thus feasible. The lack of frequent contact among foreign FEWs with each other and their high turn-over rate also limited the use of respondent-driven sampling [31].

Similar to the rest of the world, sex work has increasingly shifted from brothels to EEs in Singapore. Direct sex work at registered brothels in the designated red-light districts is regulated, while soliciting beyond these brothels is illegal [7, 8]. A study on foreign FEWs in Singapore showed that consistent condom use ranged from 37.9% for oral sex to 51.9% for vaginal sex with paid partners in 2008 [3]. This was in contrast to 97.0% for registered brothel-based FSWs in 2013, of whom the majority was also foreigners [32]. Among the different nationalities, the Thai (58.8%) and Vietnamese (56.8%) FEWs reported lower consistent condom use compared to Chinese nationals (75.9%) in 2008 [3]. This further decreased in 2015 where consistent condom use for vaginal sex with paid partners was 38.0% for the Vietnamese and 42.4% for the Thai [8]. Given that HIV/STIs spread rapidly when sex workers engage in unsafe sex and cannot access preventive services [33], we have chosen to target the Vietnamese and Thai FEWs who engaged in paid or casual sex in Singapore.

In 2016, we completed a quasi-experimental trial which demonstrated the effectiveness of a comprehensive sexual health promotion programme in increasing condom use and reducing the incidence of STIs among Thai and Vietnamese FEWs in Singapore. The outcome evaluation of the trial has been published elsewhere [34]. This paper described the needs assessment phase before implementation of the intervention where the socio-organisation, sexual risk behaviours and access to health services of foreign FEWs in Singapore were explored. We also highlighted how qualitative inquiry, census enumeration technique and community-based engagement approaches were used to gain access and to develop a culturally appropriate STI prevention intervention.

## Methods

### Methodology overview, roles of research team and time period

**Fig. 1:**
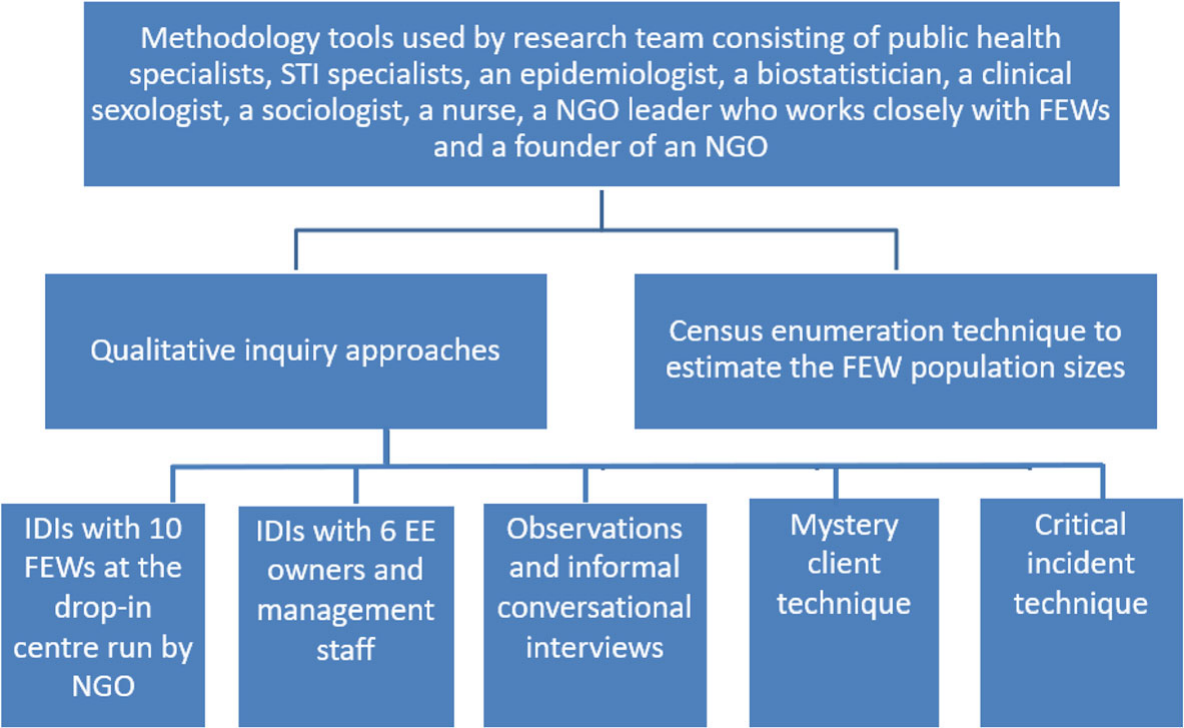
Overview of qualitative inquiry approaches and population size estimation technique used

We used (i) various qualitative inquiry approaches such as IDIs, observations, informal conversational interviews, mystery client and critical incident technique and (ii) an estimation size technique, i.e. census enumeration method to gather information about the foreign FEWs. This is summarised in Fig. 1**.** The qualitative inquiry approaches were conducted by the Principal Investigator (PI: MLW), first (RBTL) and second (ONYC) authors who have been trained in qualitative research. The first (RBTL), second (ONYC) and third (DKTT) authors supervised by the fourth author (HHL), who was part of the core writing team of the participant manual for most at risk size estimation workshops by the University of California, San Francisco and UNAIDS, estimated the population size. This needs assessment phase took place for a year from March 2014 to February 2015. The data was subsequently used to inform an intervention trial which has been registered with ClinicalTrials.gov (NCT02780986).

### IDIs with FEWs at the drop-in Centre run by NGO

The PI who has conducted research on brothel-based FSWs for more than 2 decades [35, 36] and foreign FEWs [3] used her social networks to reach out to non-governmental organisations (NGOs). These included Action for AIDS (AfA) and Humanitarian Organisation for Migration Economics (HOME) that have been conducting outreach activities for FEWs in Singapore. We visited the women’s health centre, a drop-in centre for education and health-related services for migrant women run by HOME. On an average week, about 40 FEWs visited the centre to take a break from soliciting, to meet their peers over drinks and snacks, to use the foot massager machine or to get condoms and lubes. We conducted IDIs with 10 Thai and Vietnamese FEWs.

### IDIs with EE owners and management staff

Through the social networks of NGOs, we visited EEs and in the process were introduced to some EE owners who eventually led us to the President of the Singapore Nightlife Business Association (SNBA). The SNBA is an association formed by leaders from all sectors of nightlife as a representative body to coordinate the industry’s input on all relevant matters that affect the industry. We conducted IDIs with 6 EE owners and management staff.

### Observations and informal conversational interviews

We conducted systematic and unobtrusive naturalistic observations at the study sites where the Vietnamese and Thai FEWs congregated. This served to better understand the operations of the EEs and FEWs, including interactions between FEWs and heterosexual men patronising EEs. We spoke to some Vietnamese who worked in the cafes at the study site to find out the avenues to access this population for health education and other STI prevention activities. The conversation was carried out by one of our Vietnamese research team member.

We observed that there were a few Traditional Chinese Medicine (TCM) and general practitioner (GP) clinics among the EEs in the Vietnamese FEWs’ study site and their operating hours extended into the night time. One TCM clinic provided treatment of sexual dysfunction. Through informal conversational interviews with the TCM practitioners and GPs, we soon learnt that they had in-depth information regarding FEWs and their social networks as most of them had worked in the area for decades. They subsequently introduced us to the agents who were involved in recruiting females from Vietnam for indirect sex work and helping them with the application of social visit passes.

### Mystery client technique

The first author (RBTL) used the mystery client technique, a special form of participant observation, in which he took the role of a heterosexual man patronising EE in the natural setting. This technique was previously used by the PI on foreign FEWs to determine the prevalence of indirect sex work in EEs in Singapore in 2008 [3]. During the encounters, the FEWs were friendly, and they proceeded to the table where the first author was seated to engage in small talks and to persuade him to purchase alcoholic drinks for them. These FEWs could converse in Mandarin or simple English. Whenever they hinted the possibility of paid sex through their body language (e.g. holding the first author’s hand and sitting on his lap) and suggested to the first author that special services were available, he would find an excuse as an exit strategy to leave the EE.

### Critical incident technique

Using the critical incident technique, we observed the movements of a few Vietnamese and Thai FEWs who were likely to engage in paid sex to help us understand their behaviours and movements. Vietnamese FEWs often stayed and worked within the study site. This was suggested by our observations that newly arrived Vietnamese FEWs (with their luggage) made their way to the upper levels of certain shop houses in the study site. We also observed the movements of these Vietnamese and Thai FEWs, including their interactions with men patronising the EEs, up to the time when they left the EE together.

### Qualitative data analysis

We conducted the IDIs using a topic guide developed a priori containing questions on FEWs’ socio-organisation, sexual risk behaviours, access to health services as well as needs and concerns. We took notes but did not audio-record the IDIs. This was to protect the identities of participants. The observations were also made using a checklist that was developed a priori. Findings from observations, mystery client and critical incident techniques were recorded after the fieldwork and not in the study sites. The first and second authors manually coded these data and carried out thematic analysis. Key themes were identified and agreed by the research team. We categorised these themes into broad categories, namely socio-organisation, sexual risk behaviours, access to health services as well as needs and concerns. Other than using this information to inform the development of intervention programme, they also translated into strategies for fostering participation.

### Estimation of the FEW population sizes using observation and census enumeration technique

We estimated the size of FEW population at the study sites using observation and census enumeration technique [27, 28] in December 2014. Through observations, we mapped out the 2 geographical sites, Joo Chiat Road (Eastern Singapore) and Beach Road (Southern Singapore), where Vietnamese and Thai FEWs worked respectively. To create our sampling frame, we covered the 2 geographical sites and listed down all the EEs which were in operation. The EEs were then stratified into 2 groups for each site: (i) small- to medium-sized EEs occupying only one storey without private rooms and (ii) large-sized EEs occupying more than one storey with private rooms. We then selected the EEs for the census enumeration exercise using proportionate stratified random sampling. The day and time of the exercise were determined based on prior observations to coincide with peak FEW flow at the study sites. FEWs were counted by 2 separate observers at the selected EEs during the peak hours on a weekend. For each selected EE, the average count of 2 separate observers was taken and precautions were taken to prevent duplication of count. For each stratum, the average number of FEWs per EE (obtained by calculating the average FEW count from all the selected EEs) was then multiplied by the total number of EEs to obtain the sum for that stratum. The sum of the 2 strata was then calculated to obtain the final estimated size of the FEW population in that site. The assumption was that there was negligible movement of FEWs between the 2 strata at each study site which was confirmed by prior observations.

### Overview of trial design, assessment time points, intervention and comparison programme (Fig. 2)

The research design was changed from cluster randomised controlled trial to a quasi-experimental study because of insufficient comparable cluster sites for randomisation based on needs assessment findings. Using time-location sampling, we recruited 220 FEWs (115 Vietnamese and 105 Thai women) each for the comparison and intervention groups at baseline. The study sites were first used to recruit the comparison group (March to July 2015), followed by a 3-month interval period before another new batch of participants were recruited as the intervention group (November 2015 to April 2016). Both groups completed a self-administered anonymous questionnaire as well as received STI tests at baseline and at 6 weeks’ follow-up. The intervention programme comprised a (i) behavioural component which included outreach small group sessions on STI/HIV prevention, safe sex, condom negotiation and use by peer educators, (ii) biomedical component which included free testing of cervical chlamydia, cervical gonorrhoea and pharyngeal gonorrhoea, and provision of free treatment for those tested positive, and (iii) structural component which included providing access to free condoms and lubricants. The comparison programme comprised a (i) behavioural component which included outreach small group sessions on healthy diet and physical activity by peer educators, and (ii) biomedical component which was similar to the intervention.

**Fig. 2:**
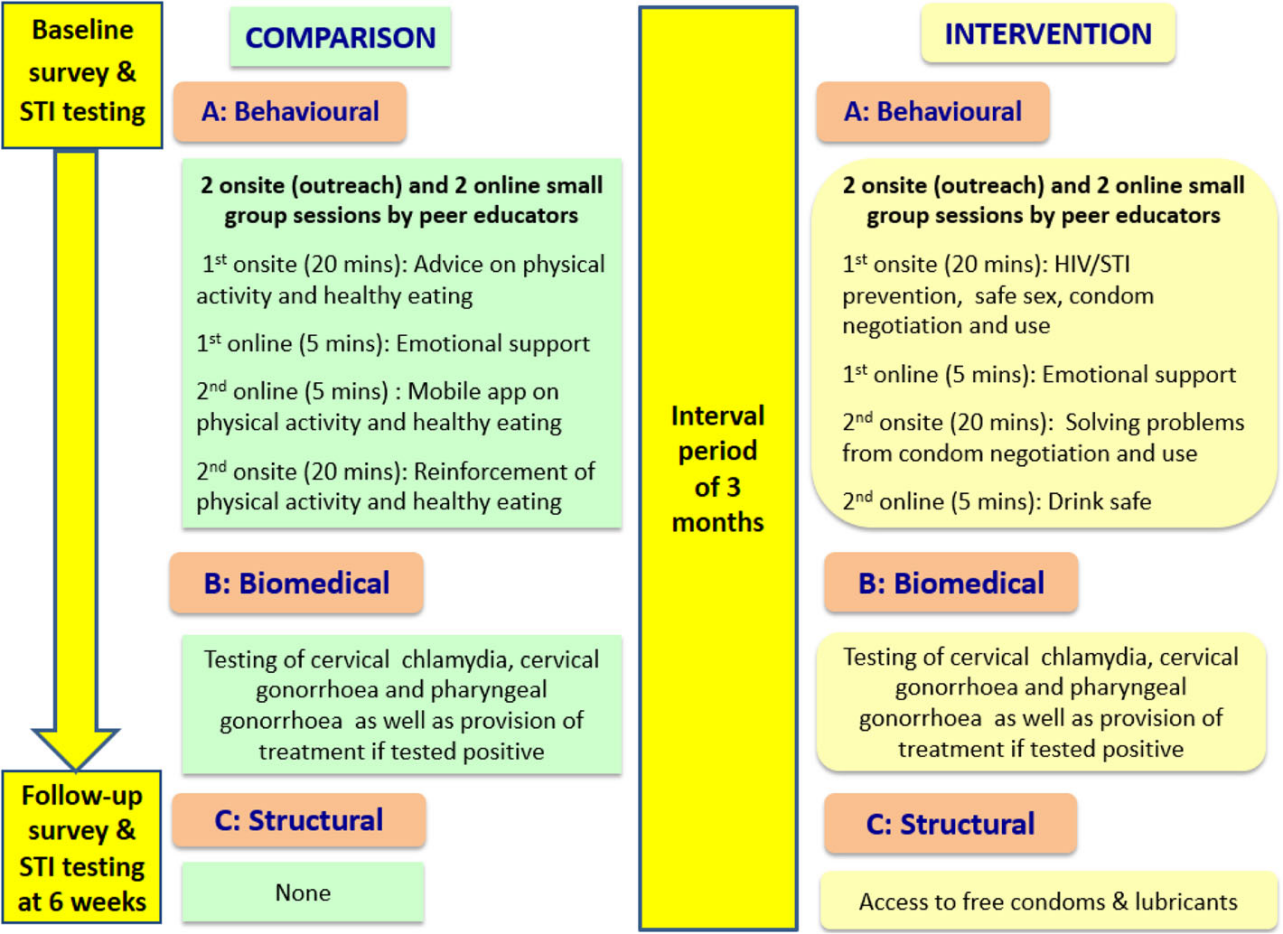
Overview of trial design, assessment time points, intervention and comparison programme

### Process evaluation of strategies

We recorded the participation and follow-up rate of the comparison and intervention group. In addition, we obtained feedback on the strategies used.

### Outcome evaluation of interventions

The primary outcome was consistent condom use for vaginal sex with casual and paid partner respectively in the past month. This was assessed by two questions using survey questionnaire at baseline and at 6 weeks follow-up, “Did you ever have vaginal sex in the past one month?” (Options of no and yes) and “If you have vaginal sex, how often did you use condoms with the specified partner?” (Options of not applicable, never, sometimes and always). Participants who gave the answer as ‘Yes’ to the first question and ‘Always’ to the second question were classified as having consistent condom usage for vaginal sex with the specified partner in the past month.

The secondary outcome was at least one incident case of STI defined as having a new episode of cervical chlamydia, cervical gonorrhoea or pharyngeal gonorrhoea at 6 weeks’ follow-up. The diagnoses of cervical chlamydia and cervical gonorrhoea were based on positive self-collected vaginal swabs using nucleic acid amplification testing (NAAT) by polymerase chain reaction (Cobas® 4800 CT/NG Test, Roche Diagnostics Basel, Switzerland). The sensitivity and specificity of self-collected swabs were equivalent to clinician-collected swabs using NAAT [37]. Throat swabs collected by healthcare personnel were used to diagnose pharyngeal gonorrhoea. As NAAT was not Food and Drug Administration-approved for pharyngeal screening, cultures were used instead.

### Ethics approval and participant consent

The study was approved by the National University of Singapore Institutional Review Board (approval certificate number NUS 2159). We obtained oral consent from all the participants who have agreed to participate in this study.

## Results

### Barriers and strategies in gaining access to gatekeepers and foreign FEWs

Gatekeepers consisting of agents, EE owners, “mamasans” (women who work and supervise FEWs in EEs in the Asian context) and staff of EEs such as bouncers posed as first-line barriers for us to gain access to FEWs. FEWs were also resistant to outsiders, including researchers. There was suspicion, resistance and lack of trust from both gatekeepers and FEWs as they perceived us as undercovers for the authority. In addition to cultural and language barriers, the social stigma and discrimination associated with indirect sex work posed another barrier.

To gain access to gatekeepers, we identified and engaged opinion leaders who can influence the opinions, attitudes, beliefs, motivations, and behaviours of people in the EE industry [38]. These opinion leaders were selected based on the criteria that they need to be credible, trusted by their own community and motivated to act as agents of change to influence the rest of their community. They also acted as our ‘guarantor’ by reassuring the community that we did not represent the authority. We spent one year to gain the trust of the gatekeeper community and shared our goal to improve the health of the FEW community. For instance, the PI emphasised her gender, medical rather than her research background, and her strong commitment to improving women’s health when introducing herself and interacting with the opinion leaders, agents, peer educators and key informants. She shared personal success stories of FSWs who were empowered from the women health’s programmes she had implemented locally and regionally that had successfully transformed their lives [35, 36]. We gained entry to the community through a gradual process, ensuring that onerous demands were not made on the gatekeepers immediately or too quickly.

To reach out to FEWs and to build trust, we engaged in outreach activities organised by NGOs. Prior to study implementation, a female sociologist from the research team volunteered with the NGOs and actively participated in the activities organised for FEWs at the drop-in-centres. This enabled us to build rapport and trust with FEWs gradually. We also wanted to convey to FEWs that we were genuinely concerned to improve their health. Through the recommendations of NGOs and informal leaders of FEWs, we engaged peer educators to overcome cultural and language barriers. The criteria for selection were that they had previously worked or currently still working in the sex industry, had similar cultural background and spoke the same native language, and were motivated to help their own community.

### Estimated FEW population sizes at study sites (Additional file 1: Table S1)

After accounting for duplications, we estimated 376 Vietnamese and 330 Thai FEWs in the 2 geographical sites where they operated in Singapore.

### Qualitative findings to inform the development of intervention programme and translation of strategies to foster participation (Table 1)

In the context of socio-organisation, we found that majority of the FEWs were not directly employed by the EEs but came on social visit passes of 1 to 2 months duration. The Thai and Vietnamese FEWs have their own preferred venues of congregation and they usually worked alone or in small groups of 2 to 3. Their main priority was to maximise financial gains within their short stay. Sources of income included tips when they chatted, sang, danced and drank with their customers, commissions from sale of alcoholic drinks and provision of sexual services. They kept to themselves and only had a few trusted peers from their own ethnic community. We thus conducted outreach intervention in these sites where they worked but not inside the EEs. Peer educators were engaged to help in recruitment and to deliver the behavioural intervention sessions. These sessions were conducted in small groups of 3 to 4.

**Table 1:** Qualitative findings to inform the development of intervention programme and translation of strategies to foster participation

Finding	Source	How the finding has informed the development of intervention programme or has led to the translation of strategies to foster participation
Socio-organisation		
Majority of the foreign FEWs were not directly employed by EEs but came on social visit passes of 1 to 2 months duration.	IDIs with EE owners and management staff, Thai and Vietnamese FEWs	It was not feasible to conduct interventions for foreign FEWs inside the EEs.
The Thai and Vietnamese FEWs have their own preferred venues of congregation. This was particularly for Vietnamese FEWs who often stayed and worked within the study site.	IDIs with Thai and Vietnamese FEWs, observations, information from mystery client and critical incident techniques	Although foreign FEWs are hard-to-reach, they operated in well-defined EEs in geographically distinct areas in Singapore, enabling us to estimate their population size using the census enumeration method, gained access to them and held community-based interventions offering outreach services in the sites where they operated.
The Thai and Vietnamese FEWs usually worked alone or in small groups of 2 to 3.		Peer educators were engaged to help in recruitment and to deliver the behavioural intervention sessions. These sessions were conducted in small groups of 3 to 4.
The Thai and Vietnamese FEWs kept to themselves and only had a few trusted peers from their own ethnic community.		
Sexual risk behaviours		
The reasons for non-condom use included misconceptions on the transmission and consequences of STI/HIV, low risk perception of contracting HIV/STI from paid/casual partner, lack of skills to negotiate or to persuade partner to use condom, unavailability of condoms in the EE setting and fear of the police using condom as circumstantial evidence.	IDIs with Thai and Vietnamese FEWs, corroborated with previous study finding in 2008 by the PI and another baseline survey in 2015	We incorporated topics like STI/HIV prevention, safe sex, condom negotiation and use in the behavioural intervention sessions. We also facilitated condom use by providing FEWs access to free condoms and lubricants.
Access to health services		
Foreign FEWs faced difficulties in accessing STI/HIV preventive and treatment services such as fear of identity exposure, stigmatisation, cost and language differences.	IDIs with Thai and Vietnamese FEWs	We provided free testing of cervical chlamydia, cervical gonorrhoea and pharyngeal gonorrhoea through outreach services, along with provision of free treatment for those tested positive.
Needs and concerns		
Vietnamese FEWs were keen on having accessories and peer support group information for their work. Red is perceived as a symbol of luck and used as a lucky colour for wrapping gifts in Vietnamese custom.	IDIs with Vietnamese FEWs	We handed out a red pouch with accessories obtained from sponsors (tissue paper, mini mirror, moisturiser, face-mask and mini hand fan). We also gave all the Vietnamese FEWs a red packet (containing a card with contact details of the women’s care centre run by NGO where they could meet their peers for support) signifying luck (referred to as “lì xì” in Vietnamese). The reimbursement for study participation was also placed in a red packet.
Their main concerns were identified as fear of identity exposure and arrest by anti-vice officers.	IDIs with Thai and Vietnamese FEWs	We assured anonymity of the study and did not collect any personal identifiers except for their contact numbers. A neutral third-party, who was trusted by the FEWs held all the links to this study. We also used premises trusted by FEWs to conduct the behavioural interventions, namely the shop of a peer educator, TCM clinic and empty space outside a convenience store located within the study sites.

On exploring their sexual risk behaviours, we found that the reasons for non-condom use included misconceptions on the transmission and consequences of STI/HIV, low risk perception of contracting HIV/STI from paid/casual partner, lack of skills to negotiate or to persuade partner to use condom, unavailability of condoms in the EE setting and fear of the police using condom as circumstantial evidence. These findings corroborated with previous study finding in 2008 by the PI [3] and another baseline survey in 2015 [8]. We thus incorporated topics like STI/HIV prevention, safe sex, condom negotiation and use in the behavioural intervention sessions. We also facilitated condom use by providing FEWs access to free condoms and lubricants. In the context of access to health services, we found that they faced difficulties such as fear of identity exposure, stigmatisation, cost and language differences. We thus provided free testing of cervical chlamydia, cervical gonorrhoea and pharyngeal gonorrhoea through outreach services, along with provision of free treatment for those tested positive.

Regarding their needs and concerns, we found that Vietnamese FEWs were keen on having accessories and peer support information for their work. Red is perceived as a symbol of luck and used as a lucky colour for wrapping gifts in Vietnamese custom. We thus handed out a red pouch with accessories obtained from sponsors (tissue paper, mini mirror, moisturiser, face-mask and mini hand fan). We also gave all the Vietnamese FEWs a red packet (containing a card with contact details of the women’s care centre run by NGO where they could meet their peers for support) signifying luck (referred to as “lì xì” in Vietnamese). The reimbursement for study participation was also placed in a red packet. Their main concerns were identified as fear of identity exposure and arrest by anti-vice officers. Other than assuring anonymity of the study, we did not collect any personal identifiers except for their contact numbers. The latter was needed to inform FEWs who tested positive for STIs so that we could link them up for treatment. In addition, a neutral third-party, who was trusted by the FEWs and not involved in this study, held the link to the unique serial number identifier on the STI test samples, code number on the questionnaire and contact numbers of the FEWs. We also used premises trusted by FEWs to conduct the behavioural interventions, namely the shop of a peer educator, TCM clinic and empty space outside a convenience store located within the study sites.

### Community-based engagement and development of culturally appropriate intervention materials

FEWs and peer educators were also invited to provide feedback and suggestions on health communication messages, intervention materials and questionnaires. This resulted in health communication messages that were non-stigmatising, acceptable and understandable by FEWs. For example, condoms were referred to as raincoats in the intervention materials. In the video on various techniques to put on condoms in a pleasurable manner for their male partners, bananas and oranges were used to represent penis and breasts respectively. Intervention materials such as condom tins with attractive designs and a web portal on women’s health related issues (including sexual health) that were of interest to FEWs were also developed.

### Process evaluation of strategies

The participation rate was 90.3%, while the follow-up rate was 70.5% for the comparison and 66.8% for the intervention group. The engagement of peer educators was well accepted by FEWs, and it was common to receive comments such as “I only trust her, ...she understands what I am saying, she used to do the same work as me, she is safe...”. In addition, the flexibility of the research team to change the intervention venues according to FEWs’ concerns and preferences met with approval by FEWs, as we received comments such as “…. her shop as a site for education….… is good and safe..., we know her, her shop is near to the club.”

### Outcome evaluation of interventions

There was a significant increase in consistent condom use for vaginal and oral sex with paid and casual partners; with a corresponding reduction in STI incidence over a 6-weeks follow-up period. At follow-up, the intervention group (75.0%) was more likely than the comparison group (41.7%) to report consistent condom use for vaginal sex with paid partners (*p* < 0.001). A similar effect change was observed for casual partners (intervention 75.3%, comparison 42.2%, *p* value < 0.001). Table 2 shows each of the STI positivity at baseline and follow-up for the comparison and intervention group, along with the proportion who received treatment. For STI incidence at follow-up, there was a decrease in the comparison (14.8 per 100 FEWs) than the intervention group (6.8 per 100 FEWs) (p 0.03).

**Table 2:** STI positivity at baseline and follow-up for the comparison and intervention group

	Comparison group		Intervention group	
	Baseline (n = 220)	Follow-up^a^ (*n* = 155)	Baseline (*n* = 220)	Follow-up^a^ (*n* = 147)
Positive in at least one of the STIs	30 (13.6 per 100 FEWs)	23 (14.8 per 100 FEWs)	34 (15.5 per 100 FEWs)	10 (6.8 per 100 FEWs)
Breakdown by each STI tested:				
Cervical chlamydia	18	12	29	6
Cervical gonorrhoea	9	7	6	3
Pharyngeal gonorrhoea	7	4	3	2
Co-infection with 2 STIs	4	0	4	1
Proportion who received treatment:				
Received treatment among those tested positive (%)	2/30 (6.7)	2/23 (8.7)	9/34 (26.5)	2/10 (20.0)

^a^The STIs included only incident cases of cervical gonorrhoea, cervical chlamydia and pharyngeal gonorrhoea at follow-up

## Discussion

The use of various qualitative inquiry approaches has helped us better understand foreign FEWs’ socio-organisation, sexual behaviours, access to health services, needs and concerns in Singapore, thus enabling us to develop needs-based and culturally appropriate STI prevention intervention for them. The environment in which they operated, including their needs differed from brothel-based FSWs and local FEWs [39]. Though majority of brothel-based FSWs in Singapore are also foreigners, they have access to STI/HIV preventive and treatment services. Under the Medical Surveillance Scheme (MSS), they are required to receive mandatory STI/HIV prevention education and STI/HIV testing (including treatment for those tested positive) regularly [39]. In addition, there is convenient access to condoms and lubricants in all licensed brothels in Singapore [39]. Local FEWs who are present in smaller numbers than foreign FEWS are directly employed by EEs and thus operate in more stable environment compared to foreign FEWs who are mobile [3, 39]. Although they do not come under the MSS [39], they generally face less barriers in accessing STI/HIV preventive and treatment services than foreign FEWs in Singapore because of their familiarity with the local community [39].

Other than for developing intervention, the information obtained from qualitative approaches could be translated into strategies to foster participation for hard-to-reach populations. Strategies such as community engagement approach, obtaining key opinion leaders’ support, building trust and rapport used to foster participation among direct FSWs in brothels [25, 26, 40] could also be applied on foreign FEWs in Singapore. Our paper has further shown that these strategies need to be aligned with the capabilities, interests and context of the target group for them to work. For example, the Sonagachi Project was a native FSW community-led HIV prevention intervention from needs assessment to project evaluation, with assistance provided by the research team [40]. For our study, we took the lead but involved the foreign FEWs actively in the development of intervention materials, recruitment of participants and delivery of the intervention programme. Due to the foreign and mobile short-term stay status of our target group, it would be unrealistic to expect them to lead the study compared to the Sonagachi Project where the target population was mainly native brothel-based FSWs operating from fixed venues [40].

The use of qualitative approaches is not without pitfalls. A spectrum of qualitative methods is often employed within the context of the same study [20]. The chosen methods will depend on the research objectives and available resources [20]. Qualitative data collection can be time-consuming and resource-intensive, and researchers need to balance these challenges with other priorities in their studies [20]. In addition, the mystery client and critical incident techniques have raised certain ethical concerns [41, 42] such as the inability to obtain informed consent and the use of disguise or simulation to obtain information [41, 42]. Even though these techniques have been used for hard-to-reach populations such as FEWs, MSM, and disabled women affected by domestic abuse [41, 42], these ethical concerns could potentially outweigh their utility. For this study, these techniques were used to minimise harm (maleficence) to foreign FEWs so that they would not be threatened by gatekeepers when they revealed their work to researchers whom they perceived to be patrons of EEs.

The use of the census enumeration technique in our study was an important practical research tool to estimate the size of hard-to-reach populations. Knowing their numbers allowed us to better allocate our resources in developing the intervention. For example, knowledge of their peak operation period and the approximate number of foreign FEWs helped us to optimise the number of outreach workers at the right time. We believe that this information would help NGOs plan and evaluate prevention services for these foreign FEWs, as well as help policymakers make informed decisions on resource allocation for them.

The intervention programme only involved treating FEWs who were tested positive. This differed from periodic presumptive treatment (PPT) that has been increasingly used to reduce the high burden of STIs among FSWs since STIs are mostly asymptomatic in women [43]. PPT is defined as treatment of curable STIs based on sex workers’ high risk and prevalence of infection, rather than based on symptoms, signs or results of laboratory tests [43]. Future scale-up interventions involving foreign FEWs could consider the use of PPT to achieve a larger impact on STI/HIV prevention. There is evidence that PPT can reduce the high prevalence of gonorrhoea, chlamydia and ulcerative STIs among sex workers [43]. Sustained STI reductions can be achieved when PPT is implemented together with peer interventions and condom promotion [43].

### Lessons learnt in researching on foreign FEWs

Our research project which led to the implementation of a successful outreach intervention for foreign FSWs provided several insights. First, creating trust is the first step towards gaining access. This takes patience and time. We took one year before FEWs and gatekeepers viewed us as non-threatening and not representing the policing authorities. Second, community engagement and the use of bottom-up approach are crucial. As researchers of hard-to-reach populations, we should learn humbly from the gatekeepers, opinion leaders, peer educators and FEWs who are the experts in this field [44]. Third, we have to find out the real needs and cultural values of FEWs so as to develop culturally appropriate recruitment strategies. Fourth, we must be open and flexible to changing the study design to achieve a balance between achieving research rigour and gaining acceptance by the hard-to-reach populations. Fifth, rather than emphasising the years of expertise in research to gatekeepers and foreign FEWs, a more effective mode of engagement to gain their acceptance would be to use emotional appeal through sharing of personal success stories that matter to them. Finally, a transdisciplinary approach is crucial to developing STI/HIV prevention interventions for foreign FEWs. Our team consisted of public health specialists, STI specialists, an epidemiologist, a biostatistician, a clinical sexologist, a sociologist, a nurse, a NGO leader who works closely with FEWs (TTW) and a founder of an NGO (RC). We were able to work towards the common goal of gaining access and implementing and evaluating the intervention among foreign FEWs; in addition to sharing skills and drawing together discipline-specific approaches.

### Limitations

This study has some limitations. First, the use of qualitative inquiry approaches is heavily dependent on the skills of researchers and could be influenced by their personal beliefs and biases. This might affect the analysis and interpretation of data. To minimise this bias, we triangulated our data through the use of various methodological approaches and met regularly to discuss and ensure that we obtained the same conclusion. Second, we only employed a single technique to estimate the population size of our target group. Our estimates could have been more robust if we have employed multiple techniques. However, we took measures to increase the rigour of the census enumeration technique by accounting for duplications, a weakness often associated with its use. Third, group membership of hard-to-reach population varied over time and thus our estimates only represented a snapshot of the FEW population sizes at one point in time, rather than the true size of the population over a longer period of time. Fourth, there might be a possibility that use of ‘peak time’ could have underestimated the number of FEWs. However, we believed that this underestimation would be small. These foreign FEWs were unlikely to take off during ‘peak time’ periods since most of them would want to maximise their financial gains given their short stay in Singapore. Most of them offered either short (i.e. oral sex or handjob within the EE premise) or intermediate length of sexual services (1 to 2 h at the nearby budget hotels), thus it is unlikely that they would be pre-occupied with one client throughout the entire ‘peak time’.

## Conclusions

The use of qualitative inquiry approaches, an estimation size technique and community-based engagement approaches have enabled us to gain access and to develop a culturally appropriate STI prevention intervention for foreign FEWs in Singapore. It has led to successful intervention implementation as well as safer sexual behaviour and STI incidence reduction. We believe that our experiences and strategies would be useful to inform other researchers, policymakers and public health practitioners in the study, prevention, and control of HIV/STIs in this hard-to-reach population.

## Additional file

Additional file 1: **Table S1.** Results of the estimated foreign female entertainment worker (FEW) population sizes using the census enumeration technique. (PDF 1054 kb)

## Abbreviations

AfA: Action for AIDS
EEs: Entertainment establishments
FEWs: Female entertainment workers
FGDs: Focus group discussions
FSWs: Female sex workers
GP: General practitioner
HIV: Human immunodeficiency virus
HOME: Humanitarian Organisation for Migration Economics
IDIs: In-depth interviews
MSM: Men who have sex with men
MSS: Medical Surveillance Scheme
NAAT: Nucleic acid amplification testing
NGOs: Non-government organisations
PI: Principal Investigator
PPT: Periodic presumptive treatment
SNBA: Singapore Nightlife Business Association
STIs: Sexually transmitted infections
TCM: Traditional Chinese Medicine
UNAIDS: Joint United Nations Programme on HIV/AIDS
USA: United States of America
