# Supplementary material for: Using qualitative and community-based engagement approaches to gain access and to develop a culturally appropriate STI prevention intervention for foreign female entertainment workers in Singapore
Source: Global Health. 2018 Apr 16;14:36. doi: 10.1186/s12992-018-0358-5 (PMC5902891; doi:10.1186/s12992-018-0358-5)
Supplement: Supplementary file 1 — Table S1. Results of the estimated foreign female entertainment worker (FEW) population sizes using the census enumeration technique. (PDF 1054 kb) [file 12992_2018_358_MOESM1_ESM.pdf]

Additional file 1: Table S1 Results of the estimated foreign female entertainment worker (FEW) population sizes using the census enumeration technique

**Site**	**Joo Chiat Road, Eastern Singapore (Vietnamese FEWs)**		**Beach Road, Southern Singapore (Thai FEWs)**	
**Low (conservative) estimate accounting for duplication count**				
Stratum	Small- and medium sized EEs	Large sized EEs	Small- and medium sized EEs	Large sized EEs
Total number of EEs in each stratum	13	7	18	6
Average number of FEWs in each stratum	23	11	4	43
Total number of FEWs in each stratum	299	77	72	258
Total number of FEWs in the site	376		330	
**High estimate without accounting for duplication count**				
Stratum	Small- and medium sized EEs	Large size EEs	Small- and medium sized EEs	Large size EEs
Total number of EEs in each stratum	13	7	18	6
Average number of FEWs in each stratum	48	13	6	54
Total number of FEWs in each stratum	624	91	108	324
Total number of FEWs in the site	715		432	
